# Cloning and Molecular Characterization of the Recombinant CVB4E2 Immunogenic Viral Protein (rVP1), as a Potential Subunit Protein for Vaccine and Immunodiagnostic Reagent Candidate

**DOI:** 10.3390/microorganisms11051192

**Published:** 2023-05-01

**Authors:** Ikbel Hadj Hassine, Jawhar Gharbi, Imene Amara, Ameera Alyami, Reem Subei, Mohammed Almalki, Didier Hober, Manel Ben M’hadheb

**Affiliations:** 1 Virology and Antiviral Strategies Research Unit, Institute of Biotechnology, University of Monastir, BP74, Monastir 5000, Tunisia; 2 Department of Biological Sciences, College of Science, King Faisal University, P.O. Box 380, Al-Ahsa 31982, Saudi Arabia; 3 Laboratoire de Virologie ULR3610, Université de Lille et CHU de Lille, 59000 Lille, France

**Keywords:** coxsackievirus B4, recombinant rVP1, molecular characterization, bioinformatics

## Abstract

The aim of the present study was, first, to clone the VP1 gene of the human coxsackievirus B4 strain E2 (CVB4E2) in the prokaryotic pUC19 plasmid expression vector then to compare it with the structural capsid proteins of the same strain using bioinformatic tools. PCR colony amplification followed through a restriction digestion analysis and sequencing process which affirmed the success of the cloning process. SDS-PAGE and Western Blotting were used to characterize the purified recombinant viral protein expressed in bacteria cells. The BLASTN tool revealed that the nucleotide sequence of the recombinant VP1 (rVP1) expressed by pUC19 highly matched the target nucleotide sequence of the diabetogenic CVB4E2 strain. Secondary structure and three-dimension structure prediction suggested that rVP1, such as wild-type VP1, is chiefly composed of random coils and a high percentage of exposed amino acids. Linear B-cell epitope prediction showed that several antigenic epitopes are likely present in rVP1 and CVB4E2 VP1 capsid protein. Additionally, phosphorylation site prediction revealed that both proteins may affect the signal transduction of host cells and can be involved in virus virulence. The present work highlights the usefulness of cloning and bioinformatics characterizations for gene investigation. Furthermore, the collected data are helpful for future experimental research related to the development of immunodiagnostic reagents and subunit vaccines based on the expression of immunogenic viral capsid proteins.

## 1. Introduction

Coxsackievirus B4 (CVB4) belongs to the genus *Enterovirus* of the subfamily of *Picornaviridae*. It has a single-stranded RNA positive-sense genome of approximately 7400 nucleotides long with a single open reading frame (ORF) flanked by untranslated regions (UTRs) at the 5′ and 3′ ends [1,2]. CVB4 is a non-enveloped small virus with about 30 nm in diameter [3,4]. The genome is translated into a single long polyprotein encoding four structural proteins, VP1 to VP4 [5,6]. The VP1 protein is the most exposed and contains major neutralization epitopes and virulence determinants. This protein has been used by several researchers to study virus evolution, serotype identification, and virulence [4,7,8]. A Diabetogenic variant of CVB4 was isolated in 1979 by Yoon and his colleagues from a 10-year-old boy. The post-mortem collected data and performed studies suggest that the boy’s diabetes was virus-induced. It is a virus related to a diabetogenic variant derived from CVB4. Tumors induced in mice by subsequent inoculation are hyperglycemia, beta-cell necrosis, and insulitis. The diabetogenic strain is known as the coxsackievirus B4 E2 strain (CVB4 E2) [9]. Polymerase chain reaction (PCR) can amplify small DNA fragments [10]. For this, a primer-based PCR method is crucial to obtain the complete cDNA of the VP1 gene. The amplified gene was subjected to cloning approaches using the pUC19 expression plasmid vector. This small vector can usually carry genes that may benefit the survival of host organisms such as antibiotic resistance [11]. In addition, the pUC19 plasmid is a high copy number plasmid, thereby improving the yield of cloned foreign DNA [12,13]. The sequencing tool is the principal method used in virology to determine and examine viruses. During this study, we employed traditional Sanger sequencing. After that, we performed a bioinformatic analysis to collect useful information for future work.

The present work highlights the usefulness of interest protein cloning and bioinformatics for gene investigation. Furthermore, the collected data are helpful for future experimental research related to the development of immunodiagnostic reagents and the development of subunit vaccines.

## 2. Materials and Methods

### 2.1. Viral Strain, Bacterial Strain, Plasmid and Growth Medium

The CVB4 E2 diabetogenic strain [9], *Escherichia coli* (*E. coli*) DH5α strain (F-φ80*lac*Z∆M15 ∆ (*lac*ZYA-*arg*F) U169 *rec*A1 *end*A1 *hsd*R17 (r_k_−, m_k_+) *pho*A *sup*E44 *thi*-1 *gyr*A96 *rel*A1 λ^-^ Invitrogen, Waltham, MD, USA) and pUC19 plasmid (Invitrogen) [14] were used. The viral strain (kindly provided by J. W. Yoon, Julia McFarlane Diabetes Research Centre, Calgary, Alberta, AB, Canada) was propagated in the HeLa cell line from BioWhittaker in Eagle’s Minimal Essential Medium (MEM; Gibco BRL, Milano, Italy) supplemented by 10% fetal calf serum (FCS; Gibco BRL), 1% (2 mM) L-glutamine (BioWhittaker, Atlanta, GA, USA), 1% non-essential amino-acids (Gibco BRL), 50 µg/mL streptomycin, 50 IU/mL penicillin (Gibco BRL), and 0.05% (2.5 µg/mL) fungizone (Amphotericin B, Apothecon, Dabhasa, India). The supernatants were collected 3 days after inoculation, clarified at 4000× *g* for 10 min, divided into aliquots, and finally stored at −80 °C. Virus titer in was stocks were determined on HeLa cells by limiting dilution assay for 50% tissue culture infectious doses (TCID_50_) using the method of Reed and Muench [15]. The bacterial strain was cultured in Luria-Bertani (LB; Invitrogen) or in LB containing 1.5% agar (Invitrogen). When required, the media was supplemented with ampicillin (Amp; 100 µg/mL).

### 2.2. PCR Amplification, Cloning and Sequencing of the CVB4 VP1 Gene

Total RNA (the genome) was completely extracted using the single-step method based on guanidium isothiocyanate or Tri-Reagent^®^ (Sigma, Taufkirchen, Germany) [16] or the EZ-10 Total RNA Mini-Preps kit (Invitrogen) according to the manufacturer’s instructions. The capsid VP1 gene of the CVB4 E2 diabetogenic strain was amplified using primers that flanked the VP1 region: CVB4E2-VP1-F: 5′-TTTTATGAAGGGCCAACAGAG-3′ (nucleotides 2440–2460, relative to strain CVB4 E2) and CVB4 E2-VP1-R: 5′-CTCGGTGGTGACAGCCTCAA-3′ (nucleotides 3251–3270, relative to strain CVB4E2). A pre-denaturation for 3 min at 94 °C was applied then followed by 35 cycles of amplification consisting of 1 min denaturation at 94 °C, 1 min annealing at 54 °C, and 1 min extension at 72 °C. A final extension step was performed at 72 °C for 10 min. Another couple of primers were used to add restriction sites to the VP1 gene: 5′-TATCGAATTCTTTTATGAAGGGCCAACAGAG-3′ and 5′-TATCGGATCCCTCGGTGGTGACAGCCTCAA-3′. The underlined sequences represent *Eco*RI and *Bam*HI restriction sites, respectively. To amplify VP1 containing the restriction sites, an initial denaturing step of 3 min at 94 °C was performed and subjected to 35 cycles of denaturation at 94 °C for 1 min, annealing at 65 °C for 1 min, and an extension at 72 °C for 1 min, followed by a final extension step of 10 min at 72 °C. Then the PCR products were verified on a 2% agarose gel and stained with ethidium bromide. The amplified VP1 band was digested with *Eco*RI and *Bam*HI (TaKaRa, Kusatsu, Japan), cleaned up using “EZ-10 Spin Column DNA PCR Products Purification” (BIO BASIC, Singapore) and ligated using a T4 DNA ligase (Roche, Basel, Switzerland) with the prokaryotic expression vector pUC19, previously digested with the same restriction enzymes. The competent *E. coli* DH5α cells were previously prepared by the calcium chloride method as described elsewhere [17]. The recombinant clones resulted from the transformation of the ligation products in the competent cells selected by the PCR colony. For this, each colony was removed from LB plates supplemented with Amp using a sterile tip and dissolved in the appropriate PCR mixture. The insert-specific primers were used. After amplification of 40 cycles, using the same PCR protocol previously applied, the PCR products were visualized by a 1% agarose gel electrophoresis. The recombinant plasmid pUC19-VP1 purification by an “EZ-10 Spin Column Plasmid DNA Kit” (BIO BASIC) was followed by restriction digestion with 15 units of *Eco*RI and *Bam*HI (TaKaRa) for 2 h at 37 °C, and the recombinant VP1 (rVP1) was further sequenced using AB PRISM^TM^ 310 Genetic Analyser (Applied Biosystems, Waltham, MD, USA), according to the Sanger method [18].

### 2.3. Preparation of Cell Lysates for SDS-PAGE

For the preparation of crude cell extracts, 1.5 mL of each grown broth cultures from samples incubated overnight were taken in separate 2 mL Eppendorf tubes and centrifuged at full speed (18,000× *g* at 4 °C) for 1 min to obtain a pellet. The supernatant was discarded and the pellet was resuspended in 100 μL of 10% Sodium dodcyl sulphate (SDS) and refrigerated (−20 °C) for 5–10 min to lyse the cells. The same sample incubated overnight post induction was repeatedly frozen and thawed several times after SDS treatment. After thawing the samples were again centrifuged at full speed for 1 min. Fifty μL of the clear supernatant was taken from each tube and placed respectively into four new 2 mL Eppendorf tubes. This was followed by the addition of 50 μL of 2 × Laemmli buffer containing loading dye to each tube and mixed by pipetting. After heating for 10 min at 95 °C, the samples (10 μL each) were loaded in separate wells in 15% SDS-Polyacrylamide gel.

### 2.4. SDS-PAGE and Western Blot Analysis of rVP1 Subunit Protein

Protein samples were separated and analyzed in SDS-PAGE as described by Laemmli (1970). This was performed at room temperature in 15% polyacrylamide gels, using the Mini-Protean tetra cell 4-gel hand casting system (BIO-RAD, China). Ten μL of the sample was loaded in each well along with 5 μL of pre-stained protein ladder (10–250 kD). The gel was run at 25 mA for approximately 2 h, stained with Coomassie Blue staining solution and distained with distain solution (40 mL methanol added to 7 mL glacial acetic acid and 53 mL distilled water to make a final volume of 100 mL), until the gel background was clear. Recombinant CVB4 Viral Protein (rVP1) was resolved on 15% SDS-PAGE and transferred onto nitrocellulose membrane (0.45 μm, SIGMA). After transfer, the membrane was blocked by 5% non-fat dry milk in PBS for 1 h and probed by Mouse anti-enterovirus clone 5-D8/1 (DAKO) diluted 1:500, membranes were thoroughly washed with PBS followed by incubation with the secondary antibody goat anti-mouse Ig-Peroxidase conjugate (SIGMA) with 1:5000 dilution. The membranes were developed with a 4-Chloro-1-Naphthol Solution or by chemiluminescence and colorimetric detection kit (BIO-RAD). All the dilutions were realized with the blocking solution.

### 2.5. Purification of rVP1 Subunit Protein

The pellet obtained from the recombinant bacterial culture was treated with lysis CelLyticB buffer (SIGMA), as per the protocol. Then batch purification of recombinant rVP1 subunit protein from the *E. coli* DH5α strain was carried out under native conditions using Ni-NTA affinity chromatography (QIAGEN, Hilden, Germany). The supernatant was carefully removed and 10–15 μL was checked for the presence of the concerned protein by SDS-PAGE analysis. The supernatant was subjected to purification using Ni-NTA affinity chromatography. One mL of 50% Ni-NTA slurry was added to 4 mL cleared lysate and mixed gently by shaking it at 4 °C for 60 min. The lysate–Ni-NTA mixture was loaded into a column with the bottom outlet capped. The bottom cap was removed and column flow-through was collected. The flow-through was saved for the SDS-PAGE analysis. The column was washed twice with 4 mL wash buffer and the collected wash fractions were also analyzed using SDS-PAGE. One mL elution buffer was used to elute the protein four times. The purity of the recombinant protein rVP1 in different fractions was checked by electrophoresis on 15% SDS-PAGE gel after staining with Coomassie brilliant blue stain.

### 2.6. Bioinformatic Analysis

For sequences homology and conserved domains analysis, NCBI BLASTN (https://blast.ncbi.nlm.nih.gov/Blast.cgi?PAGE_TYPE=BlastSearch, accessed on 15 January 2023) and Conserved Domain Database (CDD) (https://www.ncbi.nlm.nih.gov/Structure/cdd/wrpsb.cgi, accessed on 15 January 2023) [19] were used. For further proteomic analysis, the nucleotide sequence was converted to an amino-acids (AA) format by the EMBOSS Seqret tool (https://www.ebi.ac.uk/Tools/sfc/emboss_seqret/, accessed on 15 January 2023). The ProtParam tool (https://web.expasy.org/protparam/, accessed on 15 January 2023) was utilized to analyze the physicochemical properties of the rVP1 and CVB4 VP1-VP4. The properties include the relative molecular weights, the atomic composition of VP proteins’ AA, molar extinction coefficient, theoretical isoelectric point (Pi), hydrophilic parameters, instability index (II), and so on [20]. SignalP-4.0 server (http://www.cbs.dtu.dk/services/SignalP-4.1/, accessed on 15 January 2023) was used for predicting the signal peptides [21]; NetPhos3.1 server (http://www.cbs.dtu.dk/services/NetPhos/, accessed on 15 January 2023) was utilized to determine phosphorylation sites [22,23]. InterProScan (https://www.ebi.ac.uk/interpro/, accessed on 15 January 2023), and SMART (http://smart.embl-heidelberg.de/, accessed on 15 January 2023) were employed for the analysis of the structural and functional domains [24,25]. The secondary structure of each VP protein and the solvent accessibility of its AA residues were predicted by SOPMA (https://npsa-prabi.ibcp.fr/cgi-bin/npsa_automat.pl?page=/NPSA/npsa_sopma.html, accessed on 15 January 2023), and Predictprotein (https://www.predictprotein.org/, accessed on 15 January 2023) websites. Secondary structure prediction is useful for 3D structure prediction [26]. Protein modeling was determined by the automatic mode of SWISS-MODEL (https://swissmodel.expasy.org/, accessed on 15 January 2023), and model structures were visualized using RasMol 2.7.5 Software (http://www.rasmol.org/software/RasMol_2.7.5/, accessed on 15 January 2023). BepiPred-2.0 was used to predict β-cell epitopes of the proteins (http://www.cbs.dtu.dk/services/BepiPred/, accessed on 15 January 2023).

## 3. Results

### 3.1. PCR Amplification, Cloning, and Sequencing of the rVP1 Gene

**Figure 1:**
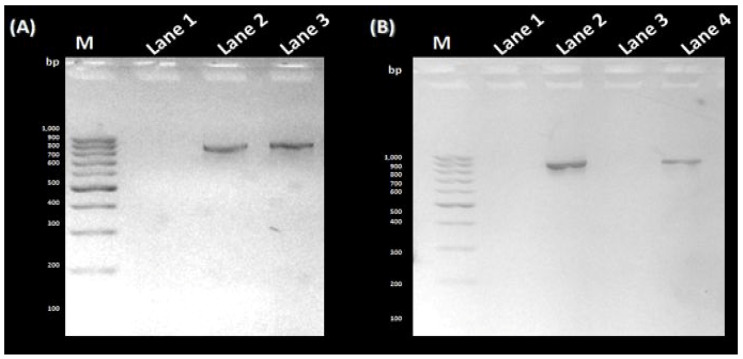
PCR amplification and Purification of the VP1 gene. (**A**) Lane M: GeneRuler 100 bp DNA Ladder (ThermoFisher, Waltham, MD, USA); Lane 1: negative control; Lane 2: PCR amplification product of the VP1 gene; Lane 3: PCR amplification product of VP1 with primers containing *Eco*RI and *Bam*HI recognition sites. (**B**) Lane M: GeneRuler 100 bp DNA Ladder (ThermoFisher); Lanes 1 and 3: negative control; Lanes 2 and 4: Purification products of VP1 and VP1 flanked by the restriction enzyme sites, respectively.

**Figure 2:**
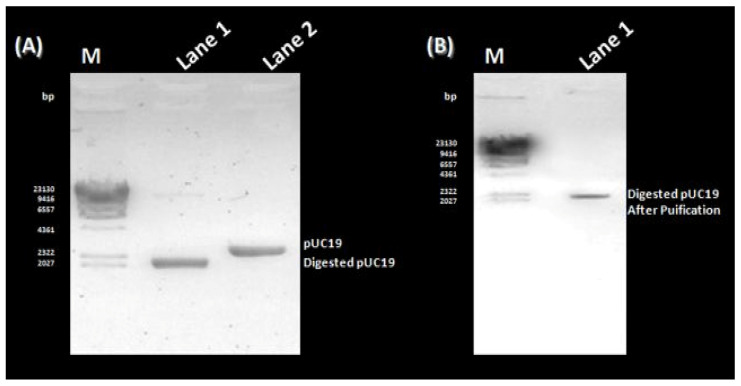
Preparation of pUC19 for the cloning process. (**A**) Lane M: Lambda DNA/HindIII Marker (ThermoFisher); Lane 1: Digestion product of purified pUC19; Lane 2: Purified pUC19 extracted from transformed bacterial cells. (**B**) Lane M: Lambda DNA/HindIII Marker (ThermoFisher); Lane 1: Purification product of the digested pUC19.

**Figure 3:**
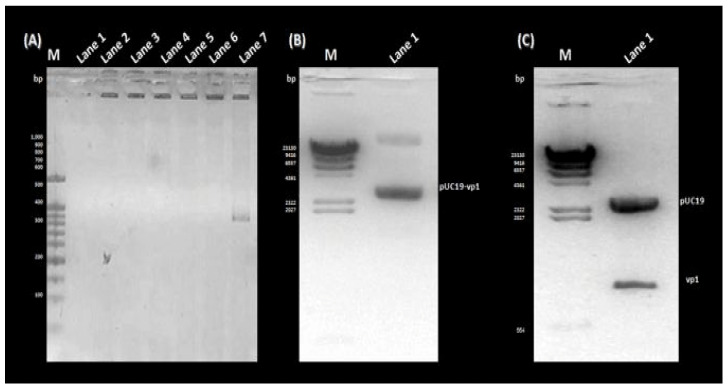
Confirmation of the cloning process. (**A**) Lane M: GeneRuler 100 bp DNA Ladder (ThermoFisher); Lane 1: Negative control; Lanes 2 to 6: PCR colony of Non-transformed colonies; Lane 7: PCR colony of a transformed colony. (**B**) Lane M: Lambda DNA/HindIII Marker (ThermoFisher); Lane 1: The extracted DNA of the same colony confirmed the cloning of VP1 in pUC19. (**C**) Lane M: Lambda DNA/HindIII Marker (ThermoFisher); Lane 1: The restriction analysis showing two bands corresponding to pUC19 and VP1.

The amplification of the VP1 gene, with or without *Eco*RI/*Bam*HI restriction sites, was carried out using the diabetogenic CVB4E2 strain. No specific band was amplified from the mock (Figure 1A, Lane 1). Whereas, a target fragment of approximately 850 bp was amplified and then purified from the positive sample (Figure 1A, Lanes 2 and 3, respectively). Before proceeding to the cloning step, a purification process is essential to remove organic and inorganic materials and undesired chemical compounds. No specific bands were observed from the mock (Figure 1B, Lanes 1 and 3). After purification of VP1 with or without restriction sites, the same bands are observed as in Figure 1 (Figure 1B, Lanes 2 and 4, respectively). To clone the DNA fragment into prokaryotic expression vector pUC19 to yield pUC19-VP1, first, pUC19 was first extracted and purified from *E. coli* transformed colonies, thus giving the expected fragment-size (Figure 2A, Lane 2). The purification was followed by restriction enzyme digestion using *Eco*RI and *Bam*HI restriction enzymes, giving a fragment with a different size (Figure 2A, Lane 1) compared to the original (Figure 2A, Lane 2). To end the cloning by a ligation reaction between the flanked and purified VP1, the digested pUC19 was also purified (Figure 2B, Lane 1). To confirm the cloning process, a PCR colony was amplified from the transformed colonies (Figure 3). As expected, the amplification of negative control (Figure 3A, Lane 1) and non-transformed colonies (Figure 3A, Lanes 2 to 6) did not show the target fragment. A fragment of approximately 850 bp was found predicting the presence of the cloned DNA fragment in the recombinant colony (Figure 3A, Lane 7). After that, the DNA from the same colony was extracted, attesting to the presence of pUC19-VP1 (Figure 3B, Lane 1). Furthermore, a restriction analysis showed two bands, each corresponding to pUC19 and the cloned VP1 (Figure 3C, Lane 1). DNA sequencing confirmed the success of the cloning process. The nucleotide sequence has been submitted in GenBank under the Accession Nb. MW193767.

### 3.2. SDS-PAGE Analysis, Western Blotting and Purification of the rVP1 Subunit Protein

**Figure 4:**
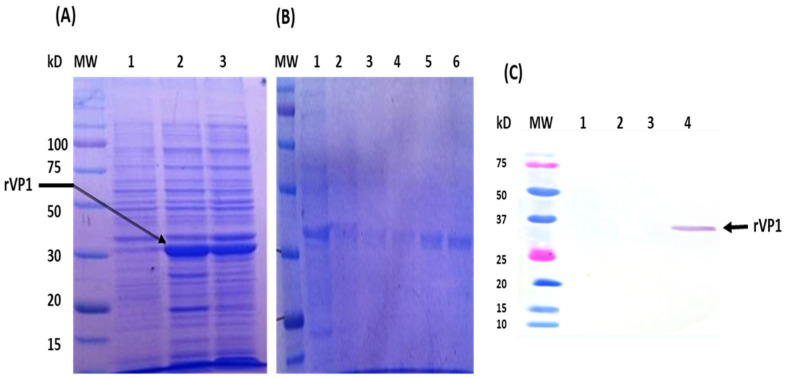
Molecular characterization of the recombinant CVB4 E2 rVP1 subunit viral protein. (**A**) SDS-PAGE analysis of the recombinant rVP1 protein. MW: Protein Weight Marker; 1: Control DH5α cells; 2–3: Transformed DH5α cells expressing VP1 gene. (**B**) Purification of recombinant rVP1 subunit protein using Ni-NTA affinity chromatography. 1: Clarified Bacteria cells expressing VP1; 2–3: Wash fractions; 4–6: Elution fractions. (**C**) Western Blotting analysis of the recombinant rVP1 protein. 1: Negative control; 2–3: Non-transformed bacteria cell lysates; 4: Transformed DH5α cell lysate expressing VP1 gene.

The molecular mass of recombinant rVP1 subunit protein, obtained through the separation of different bacterial proteins from cell lysates through using a 15% SDS-PAGE technique was estimated to be approximately 30 kD, which is very close to the expected molecular weight. Recombinant *E. coli* DH5α host cells expressing rVP1 subunit protein, subjected to overnight incubation as well as repeated freeze-thawing post-induction, lead to increased yield of recombinant VP1 subunit viral protein (Figure 4A).

After batch purification of the recombinant rVP1 recombinant protein of CVB4E2 from *E. coli* DH5α cells using Ni-NTA affinity chromatography, a relatively purified fraction of the recombinant protein rVP1 was obtained in elution fractions 4, 5, and 6, as confirmed by the 15% SDS-PAGE analysis of different fractions (Figure 4B).

The exact identity of the expressed protein was established by subjecting the recombinant *E. coli* DH5α cells to Western transfer analysis, along with negative control (non-transformed *E. coli* DH5α). Mouse anti-enterovirus clone 5-D8/1 (DAKO) conjugated with anti-mouse Ig-Peroxidase (SIGMA) directed against VP1 viral protein picked up a band at approximatiely 30 kD regions from the lanes loaded with cell lysate from the host cells transformed to express the recombinant VP1 viral protein. However, no such signal was produced in lanes from non-transformed cell lysates (Figure 4C).

### 3.3. Bioinformatic Analysis of the Sequence and Structure of the rVP1 Subunit Protein

#### 3.3.1. Bioinformatic Analysis of the Nucleotide Sequence of rVP1

**Figure 5:**
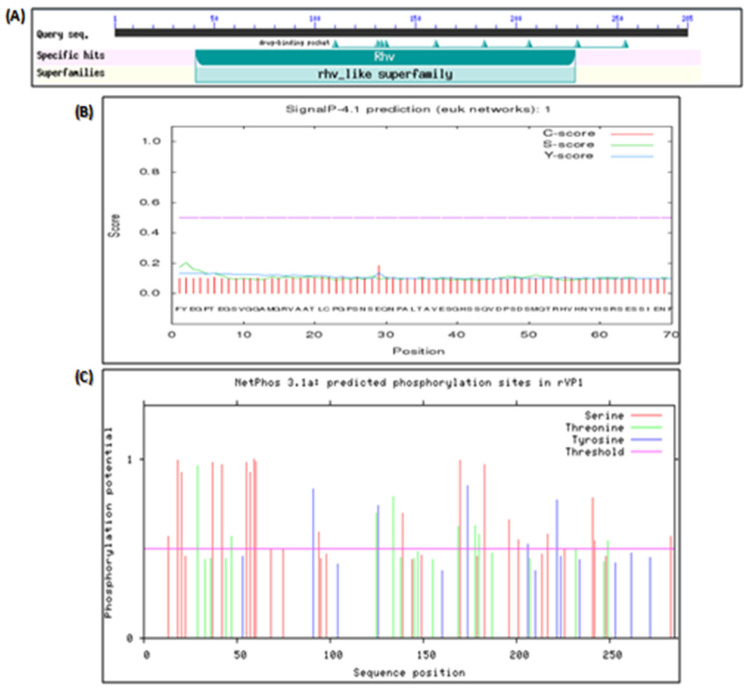
Prediction of the conserved domain, signal peptides sequence, and phosphorylation sites of the rVP1. The conserved domain, Signal peptide sequence, and phosphorylation site of rVP1 were analyzed using NCBI Conserved Domains search tool (**A**), SignalP-4.0 Server (**B**), and NetPhos 2.0 program (**C**), respectively.

The NCBI BLASTN tool showed that the nucleotide sequence of the rVP1 highly matched the target nucleotide sequence of the human coxsackievirus B4 strain E2 variant (GenBank Accession Nb. AF311939). The nucleotide sequences shared 96% identity with each other. Moreover, CDD analysis indicated that the rVP1 belonged to the rhv-like superfamily and contained a conserved domain pfam00073 (Figure 5A). As expected, the gene expression leads to a coxsackievirus capsid protein.

#### 3.3.2. Bioinformatic Analysis of the Polypeptide Sequence of rVP1

Protparam tool indicated that rVP1 expressed by the pUC19 expressing vector and VP1 protein of the Human Coxsackievirus B4E2 strain have many identical or close physicochemical properties compared to the other proteins. All the proteins are hydrophilic with a positive polarity, nevertheless, have some differences. Unlike the others, VP4 is unstable. PIs of VP3 and VP4 are less than 7 and significantly different from that of rVP1 (7.8), VP1 (8.81), and VP2 (8.83). rVP1 and VP1 have similar amino acid composition, atomic composition, and molecular weight. The analysis of CVB4 rVP1 and VP1–4 by applying different tools revealed that all the capsid proteins did not include potential signal peptide sequences (Figure 5B). However, all the proteins harbored phosphorylation sites. rVP1 includes 35 potential phosphorylation sites including 21 serine, 9 threonine, and 4 tyrosine; VP1 contains 23 potential phosphorylation sites, including 14 serine, 5 threonine, and 4 tyrosine (Figure 5C).

**Figure 6:**
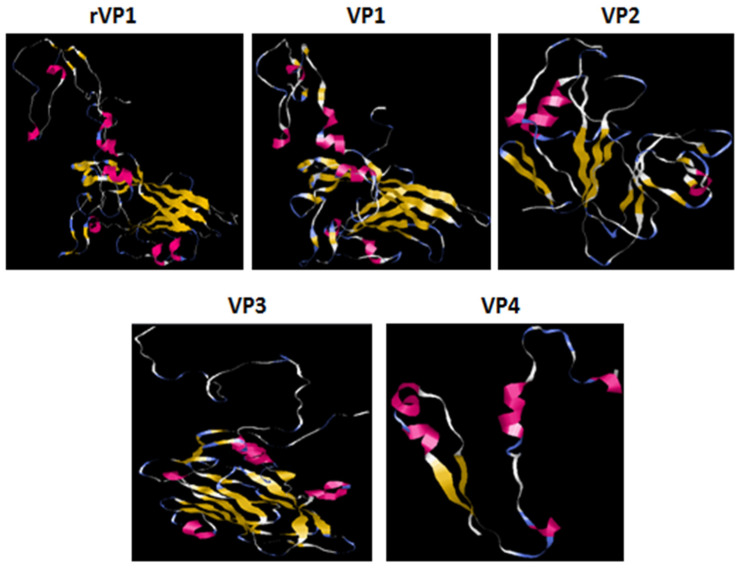
Prediction of the tertiary structure of rVP1 and CVB4E2 capsid proteins. The colored ribbons show the tertiary structure of the viral proteins. The red ones show the α helices, the yellows are the β-folding, and the white ones correspond to other residues.

**Figure 7:**
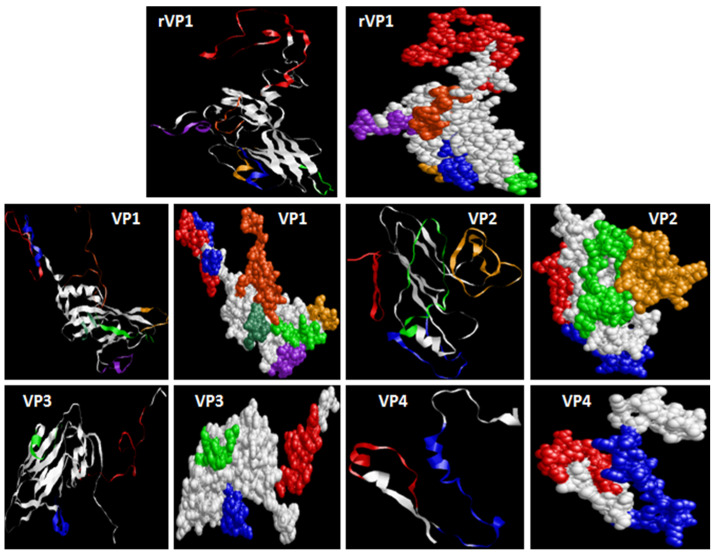
The possible B-epitope regions of the proteins and their presentation in tertiary structures. RasMol generated the tertiary ribbon and molecular surface representations. B cell epitopes are shown in red, blue, green, gold, purple, orange, and light green based on the order: AA 18–63, 83–93, 130–140, 155–168, 202–213, 258–273 of rVP1; AA 5–31, 33–50, 82–96, 127–138, 152–165, 199–209, 251–280 of VP1; AA 5–27, 38–60, 66–92, 130–165, 223–244 of VP2; AA 7–39, AA 55–67 and AA 139–150 of VP3; and AA 5–19 and AA 29–54 of VP4.

VP2–4 have a fewer number of phosphorylation sites. The subcellular location predicted by TargetP demonstrated that all the proteins could be at any extra-mitochondrial location. The prediction of the secondary structure and the solvent accessibility of AA residues are presented in Table 1. SOPMA and Predictprotein showed/revealed that random coils were the principal conformation of the capsid proteins, a higher ratio of β-folding, and lower ratios of β-turns. PredictProtein demonstrated that most of the AA residues in the VP4 protein were intermediate to solvent (83.82%). Percentages of exposed and buried AA residues in the other VP proteins were equivalent. rVP1 and VP1 have 47.65% and 45.42% of exposed AA, respectively. The protein structure homology-modeling was determined by employing the SWISS-MODEL server. The selection of templates depends on the similarity and coverage of AA sequences. Based on evaluation parameters, high standards models were applied as the tertiary structure of the VP proteins (Table 2). Model structures were observed by using RasMol 2. 7. 5 Software (Figure 6). The prediction of Linear B cell epitopes of rVP1 and VP1-VP4 capsid proteins was determined by the BepiPred server (Table 3). Among the potential linear B cell epitopes, VP4 included 2 epitopes with over 10 AA residues, respectively located at AA 5–19 and AA 29–54; VP3 contained 3 epitopes, respectively situated at AA 7–39, AA 55–67 and AA 139–150; VP2 included 5 epitopes respectively situated at AA 5–27, 38–60, 66–92, 130–165, 223–244; VP1 included 7 epitopes with over 10 AA residues, respectively located at 5–31, 33–50, 82–96, 127–138, 152–165, 199–209, 251–280; rVP1 6 epitopes 18–63, 83–93, 130–140, 155–168, 202–213, 258–273 (Table 3). The model structures were observed by using RasMol 2.7.5 Software (Figure 7).

## 4. Discussion

The aim of this study was first to clone, express, purify and characterize the VP1 gene of the CVB4E2 strain in the pUC19 plasmid and then to compare it with the structural capsid proteins of the same strain by bioinformatic tools. PCR colony amplification followed by restriction digestion analysis and sequencing process affirmed the success of the cloning process. After preparing the bacterial lysate, the expression of CVB4E2 recombinant protein was confirmed by separating different proteins in the bacterial cell lysate using the SDS-PAGE technique. The molecular mass of recombinant VP1 subunit protein, obtained by separating different bacterial proteins through a 15% SDS-PAGE technique was estimated to be approximately 30 kD, which is very close to the expected molecular weight. Bands intensity in the PAGE gel is usually proportional to the amount of protein [27]. The exact identity of the expressed protein was established by subjecting the recombinant *E. coli* DH5α cells expressing the target protein to Western Blot transfer analysis. The recombinant rVP1 subunit protein was identified by using the anti-enterovirus monoclonal antibody mAb 5-D8/1 conjugated to the anti-mouse peroxydase. This mAb picked up a band of approximately 30 kD in the elution fractions from the bacteria cell lysates. However, no such signal was produced in the non-transformed cell lysates. Purification of the recombinant rVP1 subunit protein from *E. coli* DH5α was carried out using Ni-NTA affinity chromatography. Upon purification of the recombinant rVP1 subunit protein, the purified fraction was obtained in the elution fractions 4, 5 and 6 as confirmed by 15% SDS-PAGE analysis of different fractions.

The encoded protein of rVP1 is a member of the rhv_like superfamily with a pfam00073 conserved domain. rVP1 is a Coxsackievirus capsid protein of the *Picornaviridae* family. According to the negative value of GRAVY, all the viral proteins are hydrophilic. Furthermore, they have positive polarity. However, the theoretical isoelectric point, molecular extinction coefficient, and instability index were relatively different. The theoretical isoelectric point is an important parameter to identify and purify proteins. The values of this parameter revealed that rVP1, VP1, and VP2 are basic. On the contrary, the other viral proteins are acidic. The molecular extinction coefficient is known as the absorbance of a protein at 280 nm. It depends on the number of aromatic residues, Tryptophan (Trp), tyrosine (Tyr), and cystine (Cys). The molar extinction coefficient cannot be determined by Protparam when the AA sequence does not contain aromatic residues. That is the case with the VP4 protein. If the instability index is higher than 40, it indicates that the protein is unstable, and if it is lower than 40, the protein is stable [27,28]. Unlike the other proteins, VP4 is stable. Although rVP1 and VP1 had similar molecular weights, the other proteins have significantly different molecular weights. Phosphorylation site prediction indicated that rVP1 and VP1 have more phosphorylation sites than the other viral proteins. Consequently, they are all able to affect the signal transduction of host cells and can be involved in virus virulence. The secondary structure prediction of rVP1 and VP of CV-B4 E2 principally includes random coils, also known as loops. Furthermore, unlike VP4, all the other proteins had a higher percentage of exposed AA. For several biomedical applications such as vaccine development, the determination of antigenic determinant is crucial. B-cell epitopes are hydrophilic, and have more random coils and fewer secondary structures. Furthermore, epitopes have a significantly higher ratio of exposed AA and a lesser percentage of buried AA [29]. Prediction of B-cell epitopes by applying Bepipred revealed that all the compared proteins harbored some antigenic determinants (Table 3, Figure 7). According to this, rVP1 and VP1 have the highest number of significant epitopes with similar locations. However, VP4 has a lesser number of neutralizing epitopes.

The major immunogenic protein VP1 of the CVB4E2 strain was successfully expressed in *E. coli* DH5α cells using expression vector pUC19. As a widely used method, the construction of a pUC19 vector for protein expression and purification in *E. coli* is fast, inexpensive and scalable. Ni-NTA affinity chromatography proved a rapid and excellent technique to purify the rVP1 subunit protein expressed in *E. coli*. Bioinformatic characterization proved a well-studied configuration of the expressed protein, which preserves most characteristics of the wild-type CVB4 virus proteins. Results of this preliminary study support the potential strategy to produce related subunit vaccines, and immunodiagnostic reagents based on the recombinant viral protein.

## Figures and Tables

**Table 1:** Prediction of secondary structures and solvent accessibility for the recombinant rVP1 and the CVB4E2 capsid proteins.

	rVP1	VP1	VP2	VP3	VP4
SOPMA					
α-helix	23.47%	20.07%	17.34%	17.15%	17.56%
β-folding	25.63%	23.24%	26.61%	29.29%	22.06%
β-turns	3.25%	2.11%	7.66%	5.44%	4.41%
Random coils	47.65%	54.58%	48.39%	48.12%	55.88%
Predictprotein					
α-helix	4.69%	8.45%	2.82%	8.37%	13.24%
β-folding	23.47%	26.41%	24.60%	23.43%	16.18%
Random coils	71.84%	65.14%	72.58%	68.20%	70.59%
Exposed AA	47.65%	45.42%	43.95%	42.68%	16.18%
Buried AA	7.22%	7.04%	6.05%	11.30%	-
Intermediate AA	45.13%	47.54%	50%	46.03%	83.82%

**Table 2:** Prediction of the sequences homologous to the recombinant rVP1 and the CVB4E2 capsid proteins.

	Template	GMQE	QMEAN
rVP1	1h8t.1A	0.85	−3.29
VP1	4GB3	0.78	−3.06
VP2	1mqt.1.B	0.57	−6.83
VP3	1oop.1.C	0.93	−1.90
VP4	1pov.1.A	0.57	−5.03

**Table 3:** Potential B cell epitope sites on the recombinant rVP1 and CVB4E2 capsid proteins.

Capsid	N°	Start	End	Peptide	Length
rVP1	1	7	7	E	1
	2	18	63	AATLCPGPSNSEQNPALTAVESGHSSQVDPSDSMQTRHVHNYHSRS	46
	3	83	93	SSAESNNLKRY	11
	4	30	40	QEMSSASNSDV	11
	5	155	168	PVPTSVNDYVWQTS	13
	6	202	213	WSNFSRDGIYGY	12
	7	230	234	SSPGG	5
	8	258	273	LCQYKKAKNGNFDVEA	16
VP1	1	5	31	ESVERAMGRVADTIARGPSNSEQIPAL	27
	2	33	50	AVETGHTSQVDPSDTMQT	18
	3	53	53	V	1
	4	55	61	NYHSRSE	7
	5	82	96	AESNNLKRYAEWVIN	15
	6	127	138	QEMSTATNSVVP	12
	7	152	165	PVPTSVNDYVWQTS	14
	8	176	176	N	1
	9	199	209	WSNFSRDGIYG	11
	10	227	231	SSPGGL	6
	11	251	280	PPRRLCQYKKAKNVNFDVEAVTTERASLVTT	31
VP2	1	5	27	EECGYSDRVRSITLGNSTITTQE	13
	2	38	60	WPDYLSDEEATAEDQPTQPDVAT	23
	3	66	92	LKSVKWRCSQRGGGGSSQMRCQRWVSL	27
	4	130	165	WGALTRKMRPHMVIYAEGKQQNNLNKMQSQVRLLCK	36
	5	213	215	SHY	3
	6	223	244	RERPPTFPSLQLLQALNTMACD	22
VP3	1	7	39	TPGSTQFLTSDDFQSPSAMPQFDVNPEMNIPGR	33
	2	55	67	INNLQANLKTMEA	13
	3	72	80	VRSTDEMGQ	9
	4	91	94	SSVL	4
	5	139	150	GAGAPDSRKNAM	12
	6	202	206	PAEAQ	5
	7	228	236	DTQFIKQDT	9
VP4	1	5	19	STQKTGAHETSLSAT	15
	2	29	54	INYYKDAASNSANRQDFTQDPSKFTE	26
	3	61	62	IK	2
